# Type 2 Immunity and Its Impact on COVID-19 Infection in the Airways

**DOI:** 10.3390/v15020402

**Published:** 2023-01-31

**Authors:** Prabuddha S. Pathinayake, Nikhil T. Awatade, Peter A. B. Wark

**Affiliations:** 1 School of Medicine and Public Health, The University of Newcastle and Immune Health Program Hunter Medical Research Institute, Newcastle, NSW 2308, Australia; 2 Department of Respiratory and Sleep Medicine, John Hunter Hospital, New Lambton Heights, NSW 2305, Australia

**Keywords:** COVID-19, SARS-CoV-2, type 2 immunity, airway epithelium, asthma, M2 macrophages

## Abstract

Type 2 immune responses are characterized by elevated type 2 cytokines and blood eosinophilia. Emerging evidence suggests that people with chronic type 2 inflammatory lung diseases are not particularly susceptible to SARS-CoV-2 infection. Intriguingly, recent in vitro, ex vivo research demonstrates type 2 cytokines, particularly IL-13, reduce the risk of SARS-CoV-2 infection in the airway epithelium. IL-13 treatment in airway epithelial cells followed by SARS-CoV-2 diminished viral entry, replication, spread, and cell death. IL-13 reduces the expression of the angiotensin-converting enzyme 2 (ACE2) receptor in the airway epithelium and transmembrane serine protease 2 (TMPRSS2), particularly in ciliated cells. It also alters the cellular composition toward a secretory-cell-rich phenotype reducing total ciliated cells and, thus, reducing viral tropism. IL-13 enhances Muc5ac mucin and glycocalyx secretion in the periciliary layer, which acts as a physical barrier to restrict virus attachment. Moreover, type 2 airway immune cells, such as M2 alveolar macrophages, CD4+ tissue-resident memory T cells, and innate lymphoid 2 cells, may also rescue type 2 airways from SARS-CoV-2-induced adverse effects. In this review, we discuss recent findings that demonstrate how type 2 immunity alters immune responses against SARS-CoV-2 and its consequences on COVID-19 pathogenesis.

## 1. Introduction

### 1.1. Type 2 Immunity

Type 2 immunity is mainly observed during allergic reactions, parasitic infections, and in response to envenomation. This is characterized by the production of type 2 cytokines: Interleukin-4 (IL-4), IL-5, IL-9, and IL-13, predominantly by CD4+ T cells (Th2 cells) and type 2 innate lymphoid cells (ILC2s). In the lungs, upon allergen exposure to the mucosal surface, epithelial cells produce alarmins, such as TSLP, IL-33, and IL-25, to activate tissue-resident conventional dendritic cells (cDCs) and innate lymphoid 2 cells (ILC2s). These activated cDCs then migrate into the draining lymph node and induce the differentiation of effector Th2 cells, which produce typical Th2 cytokines IL-4, IL-5, IL-9, and IL-13. Activated ILC2s also produce mainly IL-5, IL-9, and IL-13. These type 2 cytokines have various roles in regulating immune responses. The cytokine IL-4 is produced by Th2 cells, ILC2s, basophils, mast cells, and eosinophils. IL-4 regulates Th2 cell differentiation and induces isotype switching to Immunoglobulin E (IgE) production in B cells. It also suppresses type 1 immunity development, including Th1 cells and M1 macrophages [1,2,3]. IL-5 is mainly produced by CD4^+^ Th2 cells, activated eosinophils, mast cells, CD8^+^ Tc2 cells, γδ T cells, NK cells, NKT cells, and CD4^−^c-Kit^−^CD3ε^−^IL-2Rα^+^ cells in Peyer’s patches [1]. IL-5 is predominantly responsible for the recruitment and maturation of eosinophils. In asthmatic airways, IL-5 recruits eosinophils and contributes to the induction of airway hyperreactivity (AHR), and the level of IL-5 correlates with asthma severity [2,4]. Th2 cells and ILC2s are the main sources of IL-9, with minor contributions from mast cells and eosinophils. IL-9 induces eosinophilic inflammation, mast cell growth and activation, mucus hypersecretion, and AHR [5]. IL-9 is also reported to inhibit cytokine production by Th1 cells [1,3]. IL-13 is released by activated Th2 cells, ILC2s, mast cells, basophils, eosinophils, and NKT cells. IL-13 works similarly to IL-4 and activates the same signal transduction pathways to induce IgE production by B cells. It also activates and recruits mast cells and eosinophils and promotes their survival. IL-13 acts as a potent inducer of airway epithelial remodelling by inducing goblet cell metaplasia and mucus hypersecretion. It also induces AHR and disrupts the epithelial barrier function by opening the tight junctions [1,3,6,7]. Collectively, these Th2 cytokines contribute to the development of eosinophil-rich local inflammation, mucus production, bronchoconstriction, and tissue remodelling, and in chronic inflammation, this can lead to pathological airway fibrosis and thickening of the subepithelial basement membrane.

**Figure 1:**
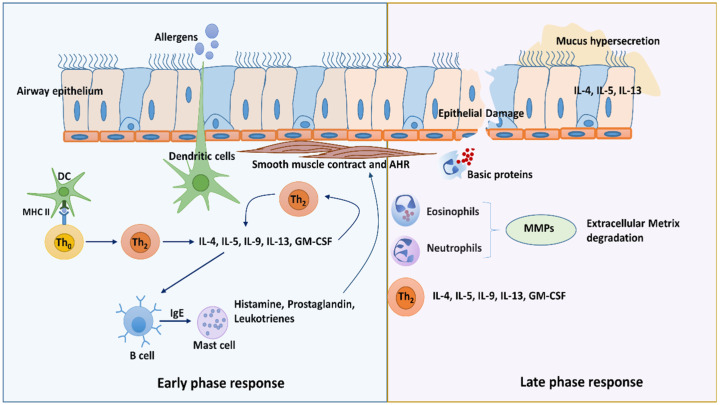
Immune reactions to allergens in asthma. At early response to allergens, dendritic cells (DCs) uptake, process, and present allergen antigens to naïve T cells (Th0). This results in the differentiation of Th0 cells to Th2 cells and secrete Th2 cytokines. These cytokines switch B cells to produce IgE and IgE recruits and activate mast cells. Activated mast cells release mediators that result in smooth muscle contraction and AHR. In the late phase, Th2 cytokines recruit a large number of inflammatory cells, such as eosinophils, neutrophils, and T cells. These inflammatory cells release mediators that cause epithelial damage, mucus hypersecretion, and degradation of extracellular matrix (ECM) and recruit more inflammatory cells into the site.

The immune responses to allergens in type 2 airways could be explained by two major phases: the early phase response and the late phase response. In the early phase, allergens are uptaken by antigen-presenting cells, predominantly by DCs, and migrate into the lung-draining lymph nodes, where they present processed antigens to naïve T cells through the major histocompatibility complex (MHC) class II. In the presence of Th2 cytokine IL-4 and the interaction of cellular receptor CD28 with CD80 or CD86 on DCs, naïve T cells differentiate into Th2 cells [8]. Th2 cells secrete cytokines, such as IL-4, IL-5, IL-9, IL-13, and granulocyte-macrophage colony-stimulating factor (GM-CSF). These Th2 cytokines play a pivotal role in modulating allergic reactions by recruiting various immune cells such as basophils, eosinophils, and mast cells. The presence of IL-4 and IL-13 promotes B cells switching IgG to IgE antibody production, which results in mast cell activation by binding into high-affinity IgE (FcεRI) receptors. Consequently, activated mast cells release mediators such as histamine, prostaglandins, and leukotrienes, which promote the induction of cellular and vascular leakage, bronchoconstriction, and the recruitment of inflammatory cells [9] (Figure 1). 

The late phase reaction to allergens occurs a few hours after and peaks at 6–9 h after allergen exposure. This reflects the action of innate and adaptive immune cells that have been recruited from the circulation and the secretion of inflammatory mediators of tissue-resident cells. These reactions also could be a consequence of mediators released by activated mast cells and antigen-stimulated T cells during the early phase [10]. The consistent recruitment of leucocytes promotes inflammation and airway epithelial damage and increases vascular permeability. For instance, elastase released by neutrophils promotes the activation of matrix metalloproteinases (MMPs) and the degradation of type III collagen. In addition, eosinophils release basic proteins and injure epithelial cells. Th2 cells recruited into the site of reaction release IL-4, IL-5, and IL-13. These cytokines cause eosinophils activation and recruitment, epithelial hyperplasia, mucus hyperproduction, and AHR [8] (Figure 1).

### 1.2. Antiviral Immunity in Type 2 Diseases

In conditions like chronic type 2 airway diseases, such as asthma, common respiratory viral infections, for instance, rhinoviruses (RVs), respiratory syncytial virus (RSV), and influenza virus, are prevalent. One of the major reasons why these viruses dominate in asthmatic airways is the impaired innate immune responses, apparently due to the Th2/Th1 imbalance due to overwhelming type 2 immune responses. With the previous research findings, it is now clear that the type 1 and 3 interferon responses against virus infections are impaired in the asthmatic airway epithelium [11,12,13,14]. In vitro experiments demonstrate that pre-treatment of airway epithelial cells with major type 2 cytokines (IL-4 and IL-13) significantly reduces the type 1 and 3 interferons in response to RV infection [15]. Further, type 2 cytokines impair interferon responses in the epithelium by reducing the interferon-responsive factor 3 (IRF3) and signal transducer and activator of transcription 1 (STAT1) signalling [15]. Another study demonstrates that the induction of the suppressor of cytokine signalling molecule 1 (SOCS1) by type 2 cytokines acts as an inhibitor of type interferon production in the airway epithelium of both mild and severe asthma patients upon RV infection [16]. Atopic dermatitis (AD), which is characterised by chronic type 2 inflammation in the skin, also shows defects in the innate immune responses and high susceptibility to cutaneous viral infections [17]. Therefore, type 2 immunity leads to an impaired antiviral response by constraining type-1-interferon-mediated innate responses in the airway epithelium.

### 1.3. SARS-CoV-2 Infection

Severe acute respiratory syndrome coronavirus 2 (SARS-CoV-2) is a highly transmissible and pathogenic coronavirus that emerged in late 2019 in Wuhan, China, and later spread all over the world causing the largest pandemic in the recent era named “Coronavirus disease 2019” (COVID-19). Coronaviruses are a diverse group of viruses that belong to the family Coronaviridae. SARS-CoV-2 belongs to the betacoronavirus 2B lineage, 79.5% homologous to SARS-CoV, which caused an outbreak from 2002 to 2004 [18]. These coronaviruses are zoonotic viruses that could infect both animals and humans.

Although some of the variants of the virus were highly pathogenic and virulent, most of them caused mild respiratory symptoms in the majority of the population. However, elderly people and people with chronic diseases were highly susceptible to the virus [19]. As the virus outbreak started in early 2020, many medical experts predicted that people with chronic respiratory diseases such as asthma and chronic obstructive pulmonary disease (COPD) would be more susceptible to the virus. However, as the virus progressed in the population, we observed that people with asthma were not overrepresented and might have some protection against COVID-19 [20]. This led researchers to investigate if there is a protective effect from type 2 immunity against COVID-19 infection and explore the underlying molecular mechanisms.

### 1.4. SARS-CoV-2 Replication Cycle

**Figure 2:**
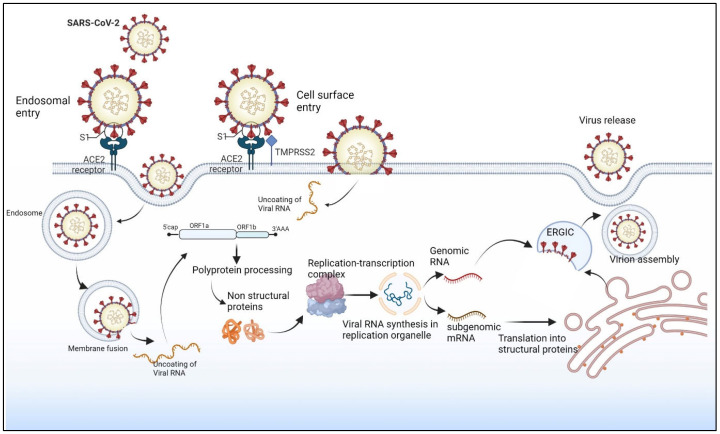
SARS-CoV-2 replication cycle. SARS-CoV-2 enters the cell via endosomal or cell surface entry. The virus attaches to the ACE2 receptor via the S1 subunit, and TMPRSS2 facilitates this process. The virus releases viral RNA into the cytoplasm and translates it into a polyprotein. Polyprotein later processes into structural and non-structural proteins. Virus assembles in ER-to-Golgi intermediate compartment (ERGIC) and releases via exocytosis. Created with BioRender.com.

SARS-CoV-2 mainly infects the upper respiratory tract and, preferentially, nasal ciliated cells [21], mucus-producing cells, and ciliated cells in the bronchial epithelium. It can also infect type 1 pneumocytes in the lung and the conjunctival mucosa [22]. However, evidence from scanning electron microscopy (SEM) images in human bronchial epithelial cells confirms that SARS-CoV-2 directly interacts with ciliated cells [23,24]. The virus mainly uses ACE2 as the entry receptor [25], while various co-receptors, including C-type lectins, DC-SIGN, L-SIGN, TIM1, AXL, and CD147, have been shown to act as alternative receptors; however, these are not as effective as ACE2 [26,27,28,29]. The attachment and fusion of the virus are mediated through the spike protein (S protein) in the virus, which gives it a crown-like appearance. The S protein encompasses two subunits, named S1 and S2. The S1 subunit attaches to the ACE2 receptor, while S2 anchors the S protein to the membrane [30]. When the virus enters through the cell surface, TMPRSS2 at the cell surface, cleaves the S2 site of the virus, facilitating virus entry through membrane fusion [31]. When the virus internalises via endocytosis, cathepsin L cleaves the S2 site and facilitates fusion with the endosomal membrane with the release of the virus genome into the cytoplasm [30]. The viral genome is flanked by 5′ and 3′ untranslated regions. At the 5′ end, the genomic RNA contains 2 large open reading frames (ORF1a and ORF1b) encoding 16 non-structural proteins (Nsps 1–16). At the 3′ end, the genome encodes the four structural proteins S, N, M, and E and nine accessory proteins, namely ORF3a, 3b, 6, 7a, 7b, 8, 9a, 9b, and 10. This process is facilitated by various viral proteases. Then, the translated structural proteins translocate into the endoplasmic reticulum (ER) membranes and transit through the ER-to-Golgi intermediate compartment (ERGIC) where interactions with N-encapsidated take place. Newly produced genomic RNA results in budding into the lumen of secretory vesicular compartments. After the virions are properly assembled, they are released from the infected cells by exocytosis (Figure 2) [32].

### 1.5. Type 2 Airway Epithelium and SARS-CoV-2 Infection

The expression level of the ACE2 receptor in the airway epithelium is thought to be directly related to the susceptibility of SARS-CoV-2 infection. We and others previously demonstrated that people with asthma have a low level of expression of ACE2 in the bronchial epithelium [33,34,35]. Using differentiated bronchial epithelial cells and biopsy samples from asthmatic patients and healthy individuals, we showed that both gene and protein expression of ACE2 is lowered in asthma. We also showed downregulated FURIN and upregulated ADAM17 gene expression in asthmatic bronchial epithelium compared to healthy controls [34]. Consistent with these findings, Kimura et al. (2020) demonstrated that IL-13 significantly reduces ACE2 expression ex vivo in bronchial airway epithelial cells [36]. They also showed that ACE2 expression was lower in Th2-high asthma compared to Th2-low asthma, and ACE2 expression inversely correlates with other markers of type 2 airway inflammation: POSTIN, SERPINB2, and CLCA1 [36]. Similarly, Jackson et al. (2020) showed that IL-13-treated nasal and bronchial epithelial cells grown in differentiated cultures express fewer ACE2 genes. They also showed that the level of allergic sensitization (IgE) in children both with or without asthma was inversely correlated with ACE2 gene expression in their airway epithelial cells [37]. Further, they demonstrated that allergen challenge in adults with allergic rhinitis, but with no history of asthma, causes reduced ACE2 expression in bronchial epithelial cells obtained by endobronchial brushings [37]. Another study, conducted using bronchial brushings and biopsies from different severities of asthmatic patients in the UK, showed no difference in the ACE2 level compared to healthy controls or between asthma severities. However, the ACE2 level was negatively correlated with Th2 gene expression and positively correlated with Th17 gene expression [38]. Together, these data, published in 2020, demonstrated a significant correlation between the status of type 2 immunity and the ACE2 expression in the airways. However, these data did not show any direct evidence of how type 2 immunity impacts SARS-CoV-2 pathogenesis.

**Figure 3:**
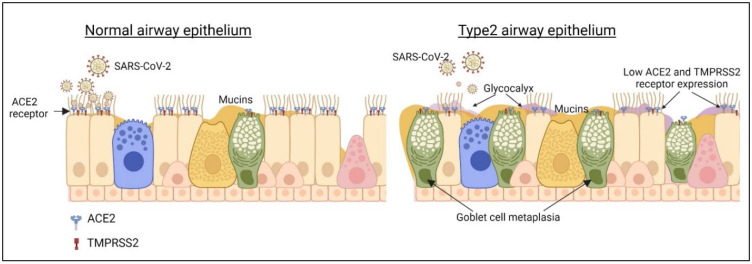
Proposed mechanism by which type 2 immunity may restrict SARS-CoV-2 virus entry and replication in the airway epithelium. Type 2 cytokines, particularly IL-13, induce goblet cell metaplasia in the epithelium, which results in a greater number of secretory cells in the epithelium and reduced ciliated cells. This results in reduced virus binding as ciliated cells stand a greater tropism towards the virus. IL-13 also reduces ACE2 expression in epithelium and TMPRSS2 in ciliated cells. IL-13 enhances mucin secretion and glycocalyx secretion in the periciliary area, restricting virus attachment. Created with BioRender.com.

A recent study, published early this year by Morrison et al. (2022), revealed that IL-13 treatment in differentiated bronchial airway epithelium significantly diminishes viral shedding and cellular damage by affecting viral entry and replication [24]. This study unveiled some of the important mechanisms underpinning type 2 immunity in SARS-CoV-2 infection. In IL-13-treated epithelial cultures, the SARS-CoV-2 mRNA and virion count were significantly reduced. The transmission electron microscope (TEM) images of infected IL-13 cultures showed minimal intracellular damage with fewer anoikis compared to IL-13-untreated infected groups. Consistent with other findings, they also exhibited that IL-13 downregulates ACE2 expression in airway epithelial cells; however, with SARS-CoV-2 virus infection, ACE2 expression was upregulated, facilitating virus binding [24]. More importantly, this study demonstrated a marked tropism of SARS-CoV-2 for ciliated cells in epithelial cell cultures, revealing that only 5% of infected cells were goblet cells and the rest were ciliated cells with virion-filled vacuoles. This would lead us to think that the altered cell composition by IL-13 in the epithelium may reduce susceptibility to infection. However, interestingly, in this study, the total number of ciliated cells was unchanged in the apical side of the cultures despite goblet cell metaplasia induced by the IL-13 [24], suggesting that cell tropism remains stable even after IL-13 treatment. With the RNA sequencing data, several gene sets involved in Keratan sulphate (KS) metabolic processes were found to be upregulated by IL-13 and this was evident by upregulated KS protein in IL-13 treated epithelial cells [24]. KS is a glycocalyx coating of the cilia and is localized to the periciliary region in the airway epithelium [39]. This thicker periciliary region may act as a physical barrier restricting viral entry along with the assistance of enhanced Muc5AC mucin production by IL-13 although mucin alone did not much affect the SARS-CoV-2 viral entry or replication (Figure 3) [24,40]. Despite reduced expression of gene network related to ciliary function in IL-13 cultures, the cilia beat frequency (CBF) was unchanged. Nevertheless, the mucociliary transport was reduced in IL-13 treated infected cultures and was associated with reduced virus spread although whether reduced mucociliary transport results in a less viral spread in human lungs is debatable [24].

Among the other upregulated gene signalling pathways in IL-13-treated epithelial cells, ion transport, glycoprotein synthesis, and protease inhibition were prominent. Signalling pathways related to cilia function/ciliogenesis and ribosomal processing were substantially downregulated. With these transcriptomic data, authors suggest that IL-13 improves mucosal defences by upregulating mucus and extracellular matrix components along with hydration and by increasing protease inhibitors with antiviral properties. Simultaneously, IL-13 hindered several mechanisms used for viral replication and spread, including protein synthesis and ciliary activity [24].

Single-cell RNA sequencing data from IL-13-treated and SARS-CoV-2-infected cells demonstrated that IL-13 treatment upregulates TMPRSS2 expression in secretory cells but decreases it in ciliated cells [40]. This could be another mechanism to explain why IL-13-treated epithelial cells result in reduced viral infections. TMPRSS2 is a serine protease that involves in SARS-CoV-2 S protein priming and facilitates viral entry. Reduced expression of TMPRSS2 in ciliated cells by IL-13 might result in reduced viral entry and diminished viral load as ciliated cells are the major target of the virus (Figure 3).

### 1.6. Effect of Immune Cells of Type 2 Airways on SARS-CoV-2 Infection

Macrophages are the most abundant immune cells in the lung. They play important roles in the maintenance of homeostasis, pathogen clearance, and immune regulation. Lung alveolar and interstitial macrophages are generally characterized and subdivided, based on their role and cytokine profile, as M1 and M2 [41]. M1 macrophages (also termed classically activated macrophages) respond to IFN-γ, lipopolysaccharide, and/or TNFα, produce proinflammatory cytokines, the direct destruction of intracellular pathogens, and promote a local Th1 environment. M2 macrophages (also known as alternatively activated macrophages) represent a more diverse phenotype and are characterised by their participation in type 2 immune responses. M2 macrophages produce anti-inflammatory cytokines, are involved in phagocytosis, and facilitate parasite encapsulation and destruction, immunoregulation, tissue remodelling, and matrix deposition [42,43]. Type 2 cytokines such as IL-4/13 activate M2 macrophages in the lung [44]. In people with type 2 asthma, M2 macrophages were found to be increased in BAL [45] and bronchial biopsy samples [46]. Typically, M1 macrophages in the lung act against viral infections by releasing type-1 IFNs and recruiting the monocytes that are necessary to mediate antiviral defence in the lungs [47,48,49]. In SARS-CoV-2 infection, a mixed induction of M1/M2 phenotype in circulating monocytes has been reported [50]. In lung tissues of severe COVID-19 patients, the alveolar macrophage phenotype showed a skew towards M1 [51]. In a human pluripotent stem-cell-derived co-culture model, both M1 and M2 have shown inhibitory effects on SARS-CoV-2 infection [51]. When infected with SARS-CoV-2, a lower number of infected hPSC-derived lung epithelial cells were observed in epithelial-M2 co-cultures compared to epithelial-M1 co-cultures implying a protective role for M2 macrophages against SARS-CoV-2 infection. Interestingly, RNA-seq analysis in these co-cultures showed elevated viral RNA in M2 macrophages compared to M1, although ACE2 and TMPRSS2 expression in hPSC-derived alveolar macrophages was undetectable. This suggests that M2 macrophages efficiently uptake viruses and infected cells via phagocytosis although they can’t be directly bind with the virus. This process may help virus clearance and further spread. In contrast, some other studies have demonstrated a higher level of ACE2 expression in primary human alveolar macrophages and suggest that alveolar macrophages can be directly targeted by SARS-CoV-2 virus [52,53]. However, a lower level of ACE2 expression is reported in M2 alveolar macrophages compared to M1 [52]. Therefore, phenotypic skewing of alveolar macrophages towards M2 may reduce the self-susceptibility to the virus due to its lower ACE2 expression and also may protect epithelial cells from virus through efficient phagocytosis. Furthermore, upon SARS-CoV-2 infection, M2 macrophages were shown to produce a significantly low level of proinflammatory mediators compared to M1 or non-activated macrophages. Instead, they showed increased phagocytosis activity and upregulated anti-inflammatory factors [51]. Since the excessive release of proinflammatory mediators in the lung upon SARS-CoV-2 infection is associated with cytokine release syndrome in severe COVID-19, reduced proinflammatory and increased anti-inflammatory mediators by M2 macrophages may avoid cytokine-induced lung tissue damage. Collectively, M2-macrophage enriched environment in type 2 airways may be less susceptible to SARS-CoV-2 infection and protect against virus-induced lung damage.

Memory T cells are important for both local and systemic protection against pathogens over a long period. Tissue-resident memory T cells (TRM cells) are characterized by the expression of the C-type lectin CD69+ and/or the integrin CD103+ [54,55]. TRM cells reside locally in non-lymphoid tissues, such as the lung, skin, and gut, where they provide frontline defence against various pathogens. These TRMs produce various kinds of cytokines, including IL-2, IFN-γ, TNF-α, and IL-17 [55]. Allergen (house dust mite) exposure in the airways triggers infiltration of both CD8+ and CD4+ TRMs into the lungs; however, only CD4+TRMs were shown to persist for a long time in lung tissues [56]. These CD4+TRMs were shown to be rapidly reactivated upon allergen exposure and associated with increased recruitment of dendritic cells into the site [56]. Another study revealed that CD4+TRMs itself is sufficient to induce mucus metaplasia, airway hyperresponsiveness, and airway eosinophil activation upon allergen reactivation [57]. Therefore, eosinophil-rich type 2 airways may harbour more CD4+TRMs, and it has been shown that CD4+-TRM are associated with asthma severity [58]. Intriguingly, apart from conventional type 2 immune functions these CD4+TRMs also exhibit protective roles against respiratory virus infections [59]. Influenza-virus-specific CD4+TRMs in mice were shown to produce IL-2 and IFN-γ in the lungs upon infection and protect mice from lethal H1N1 virus challenge [59]. Another interesting study reported that CD4+ T-cell-derived IFN-γ in the lungs is important for protection against virus infections. They showed that CD4+ T-cell-dependent signals limit the expression of the transcription factor T-bet and allow for the development of CD103(+) CD8(+) TRM cells in the airways following respiratory infection and enhance virus clearance [60]. In a recent study, a new form of CD4+ T-cell subset was found in BAL (bronchial lavage) of fibrotic lung patients. These cells are capable of releasing IL-13 (Th2) together with IFN-γ (Th1), and the authors suggest that they could be a subset of CD4+TRM as they only appear in BAL [61]. In type 2 asthma, lung fibrosis is common and may exhibit a similar subset of IL-13+ IFN-γ +CD4+ cells; however, this needs further investigation. In experimental animal models, SARS-CoV-2 infection is able to elicit both CD8+ and CD4+ TRM responses [62]. In COVID-19 convalescent patients, SARS-CoV-2 antigen-specific IFN-γ-secreting CD4+TRMs were detected. Whether a CD4+TRM-rich environment in type 2 airways provides a protective role against SARS-CoV-2 infection is unknown. Probably these existing CD4+TRMs may secrete IFN- γ and IL-2 upon SARS-CoV-2 infection and recruit CD8+ T cells into the site of infection for virus clearance. However, further studies investigating the role of CD4+TRMs in type 2 airways against SARS-CoV-2 and COVID-19 would be noteworthy.

Innate lymphoid cells (ILCs) are known to play an important role in allergic diseases, especially asthma. Activated ILC2s, a subset of ILCs, were shown to mediate type 2 responses that promote subepithelial fibrosis, airway hyperresponsiveness (AHR), smooth muscle increases, and epithelial mucus production in the airways [63]. Type 2 mediators released by ILC2s recruit eosinophils during viral lung infections upon the release of alarmins (e.g., IL-33) by damaged epithelium. ILC2s were also shown to participate in the termination of inflammatory responses and tissue repair by amphiregulin secretion [64]. During influenza virus infection, ILC2 cells were shown to promote lung-tissue homeostasis and epithelial integrity at post-virus infection and restore viral-induced tissue damage [65]. The role of ILC2 in COVID-19 is unclear. One small study found reduced numbers of circulating innate lymphoid cells, with reduced ILC2 cells associated with more severe disease [66]. Another observational study of 177 adults demonstrated that while acute COVID-19 severity was associated with overall lymphopenia, there was a specific reduction in innate lymphoid cells, and this correlated inversely with disease severity—an effect that was more marked in older individuals but was independent of both age and sex [67]. These two studies hint that the presence of ILC2 cells may provide some protection from more severe acute pneumonitis. A further observational study of 20 hospitalised patients suggested that circulating ILC2 cells that express CCR10 may play a role in recovery from acute infection with a faster resolution of inflammation and oxygenation in survivors from severe acute COVID-19 pneumonia [68]. A subset of ILC2 cells that express NKG2D, which is the activating C-type lectin-like molecule abundantly expressed by cytotoxic NK cells, was found to be elevated in patients with COVID-19 [64]. ILC2 was shown to enhance the expression of NKG2D in response to IL-18, which is also a cofactor for Th2 cell development and IgE production [64,69]. Interestingly, a significantly negative correlation was observed between COVID-19 severity and the presence of NKG2D+ ILC2s in the lungs, suggesting a protective role of this cell subset in response to the SARS-CoV-2 virus [64].

### 1.7. Microbiome in Type 2 Airways

Lung and gut microbiota play a crucial role in the development, regulation, and maintenance of healthy immune responses. Dysbiosis and dysregulation of microbiota-related immunological processes affect the onset of diseases and susceptibility to pathogenic infections. Alterations in the lung and gut microbiome have been shown to be associated with heightened respiratory virus infections such as influenza and RSV [70,71,72]. In chronic airway diseases, such as asthma, the lung microbiome is altered [73]. In type 2 eosinophilic airways, increased bacterial taxa richness and evenness (α-diversity) and decreased heterogeneity between bacterial communities (β-diversity) have been reported [74]. It is also characterized by enriched bacterial genera *Aeribacillus, Halomonas, Sphingomonas,* and *Tropheryma whipplei* and depleted *Neisseria, Bacteroides,* and *Actinomyces* [74,75]. Type 2 airway epithelial cell markers (CLCA1, SERPINB2, and POSTN) have also been negatively correlated with the total bacterial burden and relative abundance of certain taxa, including *Moraxellaceae* [76,77]. In COVID-19, the composition of the lung microbiome is shown to be distorted. In critically ill intubated COVID-19 patients, the microbial diversity was low and dominated by *Staphylococcus* and *Enterococcus* species [78]. Alpha diversity was also lower according to the severity of COVID-19 in patients, as are the relative abundances of the genera *Haemophilus* and *Neisseria* [79]. In lung tissues of deceased COVID-19 patients, *Acinetobacter* (80.70% of the total sequences) was predominantly detected. In addition, *Chryseobacterium* (2.68%), *Burkholderia* (2.00%), *Brevundimonas* (1.18%), *Sphingobium* (0.93%), and *Enterobacteriaceae* (0.68%) have been detected [80]. In another study, *Mycoplasma pneumoniae*, *Pseudomonas aeruginosa*, and *Haemophilus influenzae* were found to be the most common bacteria that were co-infected with SARS-CoV-2 [81]. Although currently there is no study demonstrating a direct relationship between the microbiome in type 2 airways and its impact on COVID-19, the altered microbiome in type 2 airways may influence initial antiviral responses to SARS-CoV-2 and the secondary bacterial and fungal infections associated with COVID-19. Further studies unveiling these aspects would be beneficial to understand the susceptibility to COVID-19 in people with chronic type 2 airway diseases.

### 1.8. Do Th2 Cytokines Worsen COVID-19?

In contrast to the beneficial role of type 2 cytokines against SARS-CoV-2, some other pre-clinical and clinical studies demonstrate that type 2 cytokines could worsen acute COVID-19. Using two different COVID-19 patient cohorts (178 patients), Donlan et al. (2021) demonstrated that plasma IL-13 is correlated with the clinical severity of acute COVID-19 [82]. Patients that are positive for COVID-19 showed elevated IL-13 levels in the plasma, and it was significantly higher in patients who needed ventilation. The plasma IL-13 level also showed an increasing trend with disease progression [82,83]. In SARS-CoV-2-infected ACE2-overexpressed k18-hACE2 transgenic mice lung samples, elevated type-2-associated genes and some histological markers, such as Ym1 (Chil3) and Resistin-like Molecule α (RELMα; Retnla), were demonstrated. The administration of anti-IL-13 antibodies drastically reduces disease severity in mice in terms of clinical score, weight loss and mortality. In another retrospective analysis using a large COVID-19 international cohort, people who had been receiving treatment for severe asthma with Dupilumab, a monoclonal antibody that blocks IL-13 and IL-4 signalling, demonstrated a lower risk of ventilation and death from COVID-19 [82]. This is also associated with reduced C-reactive protein (CRP), which is an acute-phase protein that increases during inflammation and correlates with poor outcomes in COVID-19. However, some other studies reported treatment with dupilumab was associated with reduced serum antibodies against SARS-CoV-2 [84]. With the transcriptomic analysis using anti-IL13-treated infected mouse lung samples, the enzyme Hyaluronan synthase 1 (Has1) was discovered as the most downregulated gene by IL-13 neutralization. The deposition of Hyaluronic Acid (HA) polysaccharide was found to be significantly increased in SARS-CoV-2-infected mice, specifically in the parenchyma of the lungs, and IL-13 neutralization significantly reduced the HA deposition in the parenchyma. Elevated HA in post-COVID lungs was also evident in post-mortem lung tissues of people who died of COVID-19. HA deposition followed by COVID-19 has been associated with lung fibrosis and vascular injury in patients with non-resolvable COVID-19 [85]. High levels of HA in the plasma of COVID-19 patients have been shown to directly induce endothelial barrier dysfunction in a ROCK- and CD44-dependent manner, indicating a role for HA in the vascular pathology of COVID-19 [86]. Another COVID-19 cohort in the USA also demonstrated that type 2 immune markers are associated with disease severity and continued to increase over time. The level of blood eosinophils, eotaxin-2, IL-5, and IL-13 were increased in patients with severe disease and remained higher than in patients with moderate disease [83].

## 2. Discussion

Global epidemiological data clearly demonstrate that people with mild to moderate asthma are not at an increased risk of severe acute COVID-19. In a meta-analysis, conducted using five main databases, including the World Health Organization COVID-19 database between 1 December 2019 and 11 July 2021, suggested that people with asthma were at a lower risk of acquiring COVID-19 with no increased risk of hospitalization, ICU admission, ventilator use, and mortality [87]. As 90% of children and more than 50% of adults with asthma have type 2 asthma, type 2 immunity in the lungs may play a protective role against COVID-19 infection. With the current in vitro and ex vivo data it is obvious that Th2 cytokines, particularly IL-13, reduce the risk of SARS-CoV-2 infection in the airway epithelium. Firstly, it downregulates ACE2 expression, which is the main receptor for virus binding and, consequently, reduces viral entry into the cells. Secondly, it enhances epithelial mucin secretion and glycocalyx in the periciliary layer, which acts as a physical barrier that restricts virus engagement with epithelial cells. Thirdly, the altered cellular composition towards a more secretory cell phenotype might limit virus binding sites, as SARS-CoV-2 displays a greater tropism towards ciliated cells. Although Morrison et al. (2022) demonstrated that IL-13 pre-treatment did not have any effect in reducing ciliated cells in the apical side of the differentiated airway epithelial cultures, some other studies revealed chronic IL-13 treatment (28 days) can significantly reduce the number of ciliated cells in differentiated airway epithelial cultures [88]. The discrepancy in these findings could be due to the duration of the IL-13 treatment, as Morrison et al. (2022) only pre-treated cells for 3 days [24]. This argument is further supported by the fact that IL-13 downregulates gene networks related to ciliary function and ciliogenesis in the airway epithelium. It has been reported that in asthma, the bronchial epithelium is modified and fragile, and the abnormalities include the loss of the most superficial layer of the epithelium and the destruction of ciliated cells [89]. This may impede viral entry and replication into ciliated cells. Moreover, reduced TMPRSS2 expression in ciliated cells by IL-13 may further restrict viral entry into ciliated cells. Besides these facts, Th2 cytokines may also activate mucosal defensive mechanisms independent of type I/III interferons in the epithelium, such as enhancing the secretion of protease inhibitors with some antiviral properties and restricting viral replication and spread; however, these findings need to be further investigated. In addition to epithelial cells, lung immune cells such as M2 macrophages, CD4+ tissue-resident memory T cells, and ILC2 cells, which are abundant in type 2 airways, may have a positive role in restricting viral replication and resolving virus-induced cytokine storm. However, more research towards understanding the role of these cells in SARS-CoV-2 infection in type 2 airways would be beneficial.

While clinical data from various clinical studies show that Th2 cytokines that are later elevated in the blood of COVID-19-infected people are associated with severe outcomes of COVID-19, this could be different in patients with pre-existing high type 2 immunity. People with pre-existing high type 2 profiles have altered epithelial and immune cell phenotypes that regulate immune responses differently upon virus infection and may impede virus entry and replication and inflammatory responses. These people may also have already developed negative immune regulatory mechanisms to be less sensitive to Th2 mediators to minimize lung tissue damage; however, this assumption needs to be further explored.

Collectively, these findings support the view that people with elevated type 2 immune responses may be less susceptible to SARS-CoV-2 infection and COVID-19 and have fewer adverse effects. Nevertheless, further studies towards understanding type 2 immune regulation during SARS-CoV-2 infection would be considered noteworthy.

## Data Availability

No new data were created or analysed in this study. Data sharing is not applicable to this article.
